# Tromboembolismo Pulmonar em um Paciente Jovem com COVID-19

**DOI:** 10.36660/abc.20200957

**Published:** 2020-12-01

**Authors:** Nicolas H. Borges, Thiago M. Godoy, Marcos Roberto Curcio Pereira, Rebecca B. Stocco, Viviane Maria de Carvalho Hessel Dias, Cristina Pellegrino Baena, Gustavo Lenci Marques

**Affiliations:** 1 Pontifícia Universidade Católica do Paraná CuritibaPR Brasil Pontifícia Universidade Católica do Paraná, Curitiba, PR – Brasil; 2 Hospital Marcelino Champagnat CuritibaPR Brasil Hospital Marcelino Champagnat, Curitiba, PR - Brasil

**Keywords:** SARS-CoV-2, Jovem, Tromboembolismo Pulmonar, Tríade de Virchow, COVID-19, Coronavirus-19, Assintomático

## Apresentação de Caso

**Figura 1:**
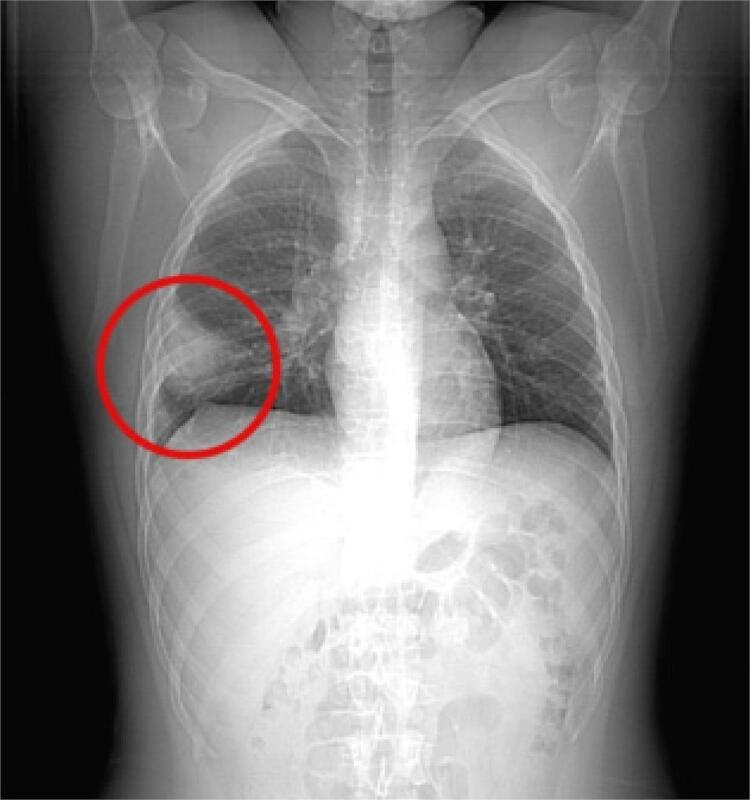
Tomografia computadorizada realizada na hospitalização, mostrando a Corcova de Humpton. Uma opacidade pulmonar bem definida com base na pleura que representa hemorragia e tecido pulmonar necrótico em uma região de infarto pulmonar causado por embolia pulmonar aguda.

Paciente do sexo masculino, 22 anos, sem comorbidades prévias e uso de medicamentos, foi encaminhado ao nosso hospital em 24/06/2020. Sem sintomas, em 12/06/2020 foi diagnosticado com COVID-19 após um teste de triagem por PCR exigido em sua empresa, e permaneceu em repouso no leito durante a maior parte de seu isolamento. Permaneceu assintomático por 11 dias, porém, em 24/06/2020, deu entrada no pronto-socorro com dor ventilatório-dependente em hemitórax direito. Os sinais vitais revelaram hipertensão (132/78 mmHg), taquicardia (127 bpm), hipóxia (SpO_2_ de 90% em ar ambiente) e febre (38,7°C). Ao exame físico, chamou a atenção a diminuição dos sons respiratórios em hemitórax direito durante a ausculta pulmonar. Os escores de estratificação de risco de Pádua e Wells foram aplicados, e os critérios indicaram um risco baixo (3 pontos) e um risco moderado (6 pontos), respectivamente. D-dímero (6,652 μg/L), Proteína C-Reativa (94 mg/L) e Troponina (119 pg/mL) estavam entre os testes laboratoriais realizados. Foi solicitada uma tomografia computadorizada (TC) de tórax ( Figura 1 ), que demonstrou a Corcova de Humpton. uma opacificação de base pleural no pulmão, mais comumente decorrente de embolia pulmonar. Além disso, também foram encontradas opacidades pulmonares em consolidação e áreas de opacidade em vidro fosco periféricas, multifocal e bilateral, associadas a espessamento septal, com pequena área de consolidação entre elas, mais acentuada no lobo inferior direito e com envolvimento pulmonar moderado (25-50%).

**Figura 2:**
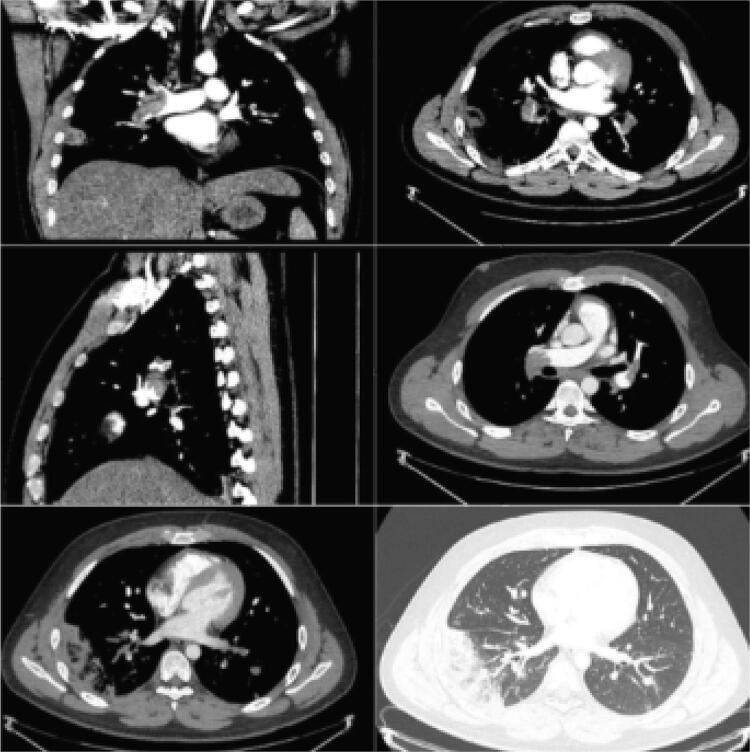
AngioTC pulmonar realizada no segundo dia, demonstrando defeitos de enchimento nas artérias pulmonares bilateralmente, reforçando o diagnóstico de tromboembolismo pulmonar agudo maciço.

Em 25/06/2020, foi solicitada uma tomoangiografia pulmonar (angioTC) ( Figura 2 ), que evidenciou defeitos de enchimento nas artérias pulmonares bilateralmente, com extensão para seus ramos superior, médio e lingual, compatível com quadro agudo maciço condição de tromboembolismo pulmonar. O paciente foi encaminhado em 25/06/2020 para a Unidade de Terapia Intensiva (UTI), hemodinamicamente estável, sendo solicitada a coleta do material de *swab* nasal e de orofaringe para SARS-CoV-2, o qual apresentou diagnóstico positivo em 26/06/2020. O tratamento foi iniciado com Ceftriaxona (2g ao dia), Azitromicina (500mg ao dia), Dexametasona (6mg ao dia) e Oseltamivir (75mg ao dia), associado à Enoxaparina (80mg ao dia) para profilaxia da trombose venosa. O paciente evoluiu com melhora progressiva. Ele teve alta da UTI para a enfermaria em 28/06/2020 e alta hospitalar definitiva em 29/06/2020, em uso de rivaroxabana (15mg BID), sendo encaminhado para futura reavaliação ambulatorial.

Após a alta, foram solicitados exames para investigação de trombofilia, incluindo: Proteína S funcional, Proteína C funcional, Homocisteína, Fator V de Leiden, Mutação do gene da protrombina, Antitrombina III, Anticoagulante Lúpico e Anticardiolipina IgM. Destacam-se o aumento da Antitrombina III (999%), a fraca presença do Anticoagulante Lúpico (1,43) e os níveis indeterminados de Anticardiolipina IgM. Além disso, foram solicitadas ecocardiografia e ultrassonografia Doppler de membros inferiores, ambas dentro dos padrões de normalidade, afastando possíveis sinais de trombose, recente ou tardia.

## Discussão

Muitos pacientes com COVID-19 têm anormalidades de coagulação que mimetizam outras coagulopatias sistêmicas associadas a infecções graves, como coagulação intravascular disseminada ou microangiopatia trombótica.^1^A coagulopatia resultante da COVID-19 pode ocorrer tanto na circulação venosa quanto arterial e está associada à liberação de citocinas pró-inflamatórias, como (IL-2, IL6, IL-7, IL-10).^2^ Os achados dos estudos mais recentes são consistentes com a estreita ligação entre trombose e inflamação, dois processos que reforçam um ao outro, pois durante a infecção por SARS-CoV-2, o endotélio é capaz de mudar para um fenótipo inflamatório responsivo após sua ativação, expressando citocinas e moléculas de adesão vascular, o que pode agravar ainda mais a tempestade de citocinas.

Essas citocinas, por sua vez, podem causar disfunções do glicocálice presente nas células endoteliais, responsáveis por criar uma barreira contra a agregação de plaquetas e células sanguíneas, contribuindo para o desenvolvimento de eventos trombóticos e endoteliais. Além disso, o estado inflamatório sistêmico também resulta em disfunção endotelial, induzindo as células afetadas a um processo de morte celular denominado piroptose.^3^ Todas essas alterações na resposta pró-inflamatória do hospedeiro, além da disfunção endotelial, também implicam em um amplo desarranjo em diversos parâmetros de hemostasia, entre os quais D-dímero,^4^ que é um potencial marcador de prognóstico e / ou mortalidade em pacientes acometidos pela doença.^5^

Apesar de não apresentar fatores de risco para complicações, o paciente deste relato desenvolveu TEP agudo maciço. Isso poderia ser explicado a partir da teoria da Tríade de Virchow, onde a disfunção endotelial, estase e hipercoagulabilidade sanguínea convergem para o desenvolvimento de processos trombóticos, entre eles, destaca-se o tromboembolismo pulmonar. O estado de hipercoagulabilidade e disfunção endotelial pode ser justificado devido à infecção viral que se refletiu em uma alteração importante no nível sérico de D-dímero (6652 µg / L), que está associado a uma maior gravidade da COVID-19; além disso, o paciente relatou que, após o diagnóstico de COVID-19, permaneceu deitado, em repouso no leito em sua residência, o que corrobora a presença de estase sanguínea pulmonar. As recomendações de isolamento pós-diagnóstico também devem ter como objetivo evitar situações que influenciem a estase sanguínea.
