# Supplementary material for: Tromboembolismo Pulmonar em um Paciente Jovem com COVID-19
Source: Arq Bras Cardiol. 2020 Dec 1;115(6):1205–7. [Article in Portuguese] doi: 10.36660/abc.20200957 (PMC8133711; doi:10.36660/abc.20200957)
Supplement: Translation

# Translation: Pulmonary Thromboembolism in a Young Patient with Asymptomatic COVID-19

**Authors:** Nicolas H. Borges, Thiago M. Godoy, Marcos Roberto Curcio Pereira, Rebecca B. Stocco, Viviane Maria de Carvalho Hessel Dias, Cristina Pellegrino Baena, Gustavo Lenci Marques

**Affiliations:** 1 Brazil Pontifícia Universidade Católica do Paraná, Curitiba, PR – Brazil; 2 Brazil Hospital Marcelino Champagnat,Curitiba, PR - Brazil

**Keywords:** SARS-CoV-2, Young, Pulmonary Thromboembolism, Virchow’s Triad, COVID-19, Coronavirus-19, Carrier State

## Case presentation

A male patient, 22 years old, with no previous comorbidities and medication use was referred to our hospital on 06/24/2020. Without symptoms, on 06/12/2020 he was diagnosed with COVID-19 after a PCR screening test required at his company and remained in bed rest during most of his isolation. He continued asymptomatic for 11 days; however, on 06/24/2020, he was admitted to the emergency department with ventilatory-dependent pain in the right hemithorax. Vital signs revealed hypertension (132/78 mmHg), tachycardia (127 bpm), hypoxia (SpO_2_of 90% at ambient air), and fever (38.7 °C). On physical examination, attention was drawn to the decrease in breath sounds in the right hemithorax during pulmonary auscultation. Padua and Wells risk stratification scores were applied, and the criteria indicated a low risk (3 points) and a moderated risk (6 points), respectively. D-dimer (6.652 μg/L), C-Reactive Protein (94 mg/L) and Troponin (119 pg/mL) were among the laboratory tests performed. A computed tomography (CT) scan of the chest was requested ( Figure 1 ), which demonstrated the presence of Hampton’s hump, a pleural-based opacification in the lung most commonly due to pulmonary embolism. Moreover, consolidative pulmonary opacities and peripheral ground-glass areas, both multifocal and bilateral, associated with septal thickening, with a small area of consolidation in between, more accentuated in the right lower lobe and with moderate pulmonary involvement (25-50%) were also found. On 06/25/2020, a computed tomography pulmonary angiogram (CTPA) was also requested ( Figure 2 ), which demonstrated filling defects in the pulmonary arteries bilaterally, extending to its upper, middle and lingual branches, compatible with massive acute pulmonary thromboembolism. The patient was transferred on 06/25/2020 to the Intensive Care Unit (ICU), hemodynamically stable, and then nasal swab and oropharynx material collection for SARS-CoV-2 was requested, which came back with a positive diagnosis on 06/26/2020. Therapy was started with Ceftriaxone (2g daily), Azithromycin (500mg daily), Dexamethasone (6mg daily) and Oseltamivir (75mg daily), associated with Enoxaparin (80mg full dose) as prophylaxis for venous thrombosis. The patient showed progressive improvement. He was discharged from the ICU to the infirmary on 06/28/2020 and on 06/29/2020 he was discharged from the hospital, using Rivaroxaban (15mg BID), being referred for future outpatient reassessment. After the discharge, tests were requested to investigate thrombophilia, including: Functional Protein S, Functional Protein C, Homocysteine, Leiden Factor V, Prothrombin gene mutation, Antithrombin III, Lupus Anticoagulant and Anticardiolipin IgM. The increase in Antithrombin III (999%), the weak presence of Lupus Anticoagulant (1.43) and undetermined IgM Anticardiolipin levels stand out. In addition, echocardiography and Doppler ultrasonography of the lower limbs were requested, which were both within normal standards, ruling out possible signs of thrombosis, either recent or late.

**Figure 1:** CT scan performed at admission, showing Hampton’s hump, a well-defined pulmonary pleural-based opacity representing hemorrhage and necrotic lung tissue in a region of pulmonary infarction caused by acute pulmonary embolism.

**Figure 2:** CTPA performed on day two, demonstrating filling defects in the pulmonary arteries bilaterally, reinforcing the diagnosis of massive acute pulmonary thromboembolism.

## Discussion

Many patients with COVID-19 have coagulation abnormalities that mimic other systemic coagulopathies associated with severe infections, such as disseminated intravascular coagulation or thrombotic microangiopathy.^1^Coagulopathy resulting from COVID-19, can occur in both venous and arterial circulation, and is associated with the release of pro-inflammatory cytokines, such as (IL-2, IL6, IL-7, IL-10).^2^The findings of the most recent studies are consistent with the close association between thrombosis and inflammation, two processes that reinforce each other since, during SARS-CoV-2 infection, the endothelium is capable of switching to a responsive inflammatory phenotype after its activation, expressing cytokines and vascular adhesion molecules, which can further aggravate the cytokine storm.

These cytokines, in turn, can cause dysfunctions of the glycocalyx present in endothelial cells, which are responsible for creating a barrier against the aggregation of platelets and blood cells, thus contributing to the development of thrombotic and endothelial events. Additionally, the systemic inflammatory state also results in endothelial dysfunction, inducing the affected cells to a cell death process called pyroptosis.^3^All these changes in the pro-inflammatory response of the host, in addition to endothelial dysfunction, also imply a wide disarray in several hemostasis parameters, among which D-dimer,^4^which is a potential marker of prognosis and/or mortality of patients affected by the disease.^5^

Despite not having risk factors for complications, the patient in this report developed massive acute PTE. This could be explained from the Virchow’s Triad theory, in which endothelial dysfunction, stasis and blood hypercoagulability converge for the development of thrombotic processes, and among them, pulmonary thromboembolism stands out. The state of hypercoagulability and endothelial dysfunction can be justified due to the viral infection that reflected in an important change in serum D-dimer level (6652 µg/L), which is associated with greater COVID-19 severity; moreover, the patient reported that after being diagnosed with COVID-19, he remained in bed rest in his residence, which corroborates the presence of pulmonary blood stasis. Post-diagnosis isolation recommendations should also aim to prevent situations that influence blood stasis.
